# Value-Based Reimbursement in Collectively Financed Healthcare Requires Monitoring of Socioeconomic Patient Data to Maintain Equality in Service Provision

**DOI:** 10.1007/s11606-018-4661-x

**Published:** 2018-09-11

**Authors:** Toomas Timpka, James M. Nyce, Isis Amer-Wåhlin

**Affiliations:** 1 Centre for Health Service Development, Region Östergötland, Linköping, Sweden; 2 Department of Medical and Health Sciences, Linköpings Universitet, SE-581 83 Linköping, Sweden; 3 Department of Anthropology, Ball State University, Muncie, IN USA; 4 Medical Management Center (LIME), Department of Women and Child Health, Karolinska Institute, Stockholm, Sweden

**Keywords:** health service administration, value-based models, socioeconomic disparity, health economy, collectively financed healthcare

## Abstract

Value-based purchasing is increasingly discussed in association with efforts to develop modern healthcare systems. These models are the most recent example of models derived from health economics research intended to reform collectively financed healthcare. Previous examples have ranged from creation of pseudo-markets to opening these markets for competition between publicly and privately owned enterprises. Most value-based purchasing models tend to ignore that health service provision in collectively financed settings is based on an insurance with political, social obligations attached that challenge the notion of free market and individualist premises which these models rest on. Central social issues related to healthcare in any modern complex society, such as inequality in service provision, can all too easily “disappear” in value-based reform efforts. Based on an analysis of Swedish policy development, we contend that management information systems need to be extended to allow routine monitoring of socioeconomic data when models such as value-based purchasing are introduced in collectively financed health services. The experiences from Sweden are important for health policy in Europe and other regions with collectively financed healthcare plans.

## INTRODUCTION

Value-based purchasing is used to maximize healthcare delivery yield by shifting emphasis from volume of services provided to quality of patient outcomes achieved.^1^ Health economists have the last five decades estimated the value of health as “gross value,” what individuals (or others acting on their behalf) would be willing to pay to acquire a certain quality of health services or “opportunity cost,” i.e., what benefits or other resources they are willing to forgo to obtain these services. To inform resource allocation decisions, these analysts have calculated the value of a health intervention in terms of incremental cost per quality-adjusted life-year (QALY) or disability-adjusted life-year (DAILY) gained. However, the present value measures may not always fully capture the health (or well-being) of patients, or incorporate individual or community preferences about the priority to be given to particular health gains, e.g., about disease severity, equity of access, or unmet need. A recent health care reform considered by Sweden’s largest health authorities also focused on value.^2^ In the simplified service purchasing healthcare models proposed, service value is determined by dividing health outcomes with costs,^3^ evidently inspired by what most Western business schools teach about management and the free market.^4^ Early critics pointed out that the core logic of these recent models may increase inequality because they penalize healthcare organizations that provide care to disadvantaged populations.^5, 6^ Other critics have argued that the introduction of business-school models has so far failed to improve health services.^7^ Further, it has been pointed out that the models lack empirical foundation, because they never have been applied and evaluated throughout any healthcare system.^8^ The question is why simplified value-based models are attractive to politicians and health service organizations in countries such as Sweden. The challenges associated with choice of key performance indicators and the risk of increasing health service inequality imply that these models require close scrutiny not just in Sweden but in all countries with collectively financed sectors of healthcare.

## VALUE-BASED HEALTHCARE REFORMS IN SWEDEN

Sweden is a society with high quality of life and an innovative knowledge-based economy underpinned by transparent and efficient institutions, reliable infrastructure, and low public debt.^9^ Further, the Swedish welfare state and public sectors has long been committed to addressing social inequality. However, over time, the evolving combination of a free-market economy with the welfare state, often referred to as the “Nordic Model”,^10^ has embraced social responsible initiatives, such as encouraging private sector competition to provide state-funded care for the elderly, private-public pensions, and a system of school vouchers to allow private and public schools to compete. Still, at first glance, a reform effort based on a free-market model does not seem to harmonize well with how Sweden has shaped its economy and healthcare system over the last 50 years.^11^

Further, there is in Sweden a general distrust of private enterprises in the public sector, fueled recently by reports of large profits being made in elementary education and the care of the elderly. Despite all this, there are at least two reasons why value-based purchasing seems to make sense and therefore has been be introduced in Sweden today. First, the reform agenda seems rational and logical in Swedish terms because it appears to work “democratically,” i.e., through a series of negotiations and discussions through which everyone involved can (theoretically) assess value for themselves.^12^ However, here some mechanisms characteristic of Swedish forms of social policy development, such as discussion, rationality, and consensus,^13, 14^ have been suborned. In other words, while the value-based models drive Swedish healthcare towards the marketplace, their rhetoric also appears to help preserve and legitimatize Sweden’s natural order of things.^15, 16^ This works because the concept of “value” as an assessment measure conceals more problematic and refractory things like inequity within itself. This is regardless of the increasing socio-demographic disparities in health outcomes emerging in Swedish society.^17^ It is therefore not surprising that the value-based models, which exclude social justice from the value concept, is entering the stage at a time when there is a large and visible increase in Sweden’s minority populations which challenges the democratic ideology that underlies the country’s social and healthcare services.

Second, Sweden’s attempt at healthcare value-based reform is validated because it originated with and is supported by elite academic networks, another cornerstone in the Swedish modern social policy. What seems to be at work here is a global tendency to reduce almost everything to a commodity whether it be ideas, things, or an agenda.^18^ Further, several of the models introduced into Sweden seem to have drawn impetus and credibility from demonstration projects associated with the US Affordable Care Act, such as Hospital Value-Based Purchasing.^19^ Again, it assumed that these marketplace models would work equally well in tax-financed service systems and that market competition can be the answer to all of a society’s existing problems.

As well, Sweden, as a nation, has been subjected to some concerted ideological marketing, which assumes that whole-scale commodification of healthcare is a good and a self-evident solution to healthcare crises anyplace in the world. There is not necessarily any element of bad faith at work here. The substitution of value for matters associated with inequality is in Sweden facilitated by the country’s historical inattention to issues associated with inequality other than unemployment.^20^ In short, these marketplace agendas make sense in Sweden because it has helped legitimatize what Sweden for at least 70 years has believed about herself.

## WHAT CAN BE DONE TO SOLVE THE PROBLEM?

Like several other European countries, Sweden is presently facing a number of healthcare challenges. The most pressing of these is that the healthcare plan is not economically sustainable.^21^ The proportion of elderly in the population increases daily as does the number of individuals suffering from multiple and chronic illnesses requiring extensive specialist care. At the same time, new, expensive drugs and treatments are being introduced at an accelerating rate, dramatically increasing costs of specialist healthcare. Progressive Swedish healthcare practitioners, for more than 50 years have attempted to limit health service inequities in Sweden’s welfare state.^22–24^ It is striking that the healthcare problems described more than half a century ago—waiting lists for specialist care, shortage of qualified general practitioners and nurses, inadequate psychiatric treatment, and less than humane care of the elderly^25^—still occur. Additionally, their causes, which these progressives identified five decades ago, remain much the same. If the concept of value-based healthcare is to make sense, given these preconditions, it must take a perspective that reflects all of the country’s population and its socio-political program rather than just be concerned with (and measure) relatively the outcome of short-term discrete clinical interventions.^26^

Clinical services provided to patients therefore need to be valued from the vantage point of the entire population in Sweden, rather than by models that compare interventions provided to individual patients. The present top causes of morbidity, both in Sweden and globally, represent chronic diseases^27^ that cannot be effectively addressed through the single “count” interventions shorn of epidemiological and social context. Health services financed through a collective insurance therefore necessitate that governance models optimize primary, tertiary prevention, instead of pinpointing discrete interventions and relying on market-based measures of value divorced from society and culture.^18^ Also in such models, care must be taken not to shift resources (further) away from society’s more disadvantaged groups. To achieve this, instead of comparing all same-type care facilities against each other, comparisons should factor in regional and socioeconomic differences. This requires a reworking of the health information systems used today in collectively financed healthcare. These systems should be able to represent not only clinical processes and quality measures^28^ but should also add cultural sensitivity to these models by including socioeconomic data from patients and service populations.^29^ If this is done and aligned with reengineering of healthcare and community services, providers servicing underprivileged populations would not be disadvantaged,^30^ and resource use across organizational boundaries would become more efficient.

## POLICY IMPLICATIONS

Health service provision in Sweden is currently open to private entrepreneurs; thus, it represents both a free and collectively financed healthcare market. Nonetheless, this is not unique to Sweden. Issues related to a smaller state budget and the resultant inequality in healthcare services seem to have fallen out of most liberal discussions on democracy like they have fallen out of the Swedish healthcare reform agenda. In Europe, for instance, attempting to find solutions to the pressing social and economic issues, such as those associated with immigration, while preserving the traditional values of solidarity and security represents a challenge. Also, just as in Swedish healthcare reform, in Europe today when it comes to social issues, only rhetoric, pragmatism, and instrumentalism seem to matter. Sadly, Reinhart was correct when he predicted 20 years ago that European healthcare management models would resemble beasts lacking ideological foundations being beset by both budgetary and medico-statistical embroilment.^31^ It is disquieting that what these instruments “forget” is precisely what makes them threaten the public and social good. The exclusion of issues related to social inequality means that the underprivileged are those who will continue to suffer the most when overly simplified healthcare models are set loose. No matter how domesticated these models may become, none of them will pay much attention to matters related to social inequality, ironically the same issues many citizens in countries with collectively financed healthcare still do not want to accept exist in their own societies. Policy measures should therefore be taken to ensure that healthcare management is augmented by information systems that include the kinds of patient data that allow for the routine monitoring of inequalities in health service provision. Only by routinely collecting and analyzing such data in management information systems, unjustified differences in healthcare access and health outcomes can be detected and controlled.

## CONCLUSIONS

Few present health value assessment frameworks simultaneously reflect the perspectives of the patient, the health plan, and society as a whole. When building any such framework, it is essential that the value construct represented there is clearly articulated. For decisions regarding societal and health plan resource allocation, including coverage and reimbursement decisions, the perspective built in should reflect, at a minimum, both acute and chronic diseases and all subpopulations who ultimately pay for care, ranging from patients and their relatives to the other insured and taxpayers.^32^ This implies that not until issues of inequality are addressed in the models marketed today to collectively financed healthcare providers, the ideal that every citizen has equal access to the socioeconomic resources of his/her country will not be established. A necessary first step to temper the side effects of efforts like value-based purchasing on underprivileged populations is to include socioeconomic data in the management information systems that support decisions regarding national health care reimbursements.
